# Endovascular Treatment of Complex Aortic Dissection. A Single Center 5 Years' Experience with 36 Patients

**DOI:** 10.31083/j.rcm2405133

**Published:** 2023-04-27

**Authors:** Ivo Petrov, Zoran Stankov, Strahil Vasilev, Iveta Tasheva, Galina Kozareva

**Affiliations:** ^1^Department of Cardiology, angiology and electrophysiology, “Acibadem City Clinic” University Hospital, 1700 Sofia, Bulgaria; ^2^Department of Diagnostic Imaging, “Acibadem City Clinic” University Hospital, 1700 Sofia, Bulgaria

**Keywords:** non-covered stents, decompression, aorta remodeling, aorta dissection

## Abstract

**Background:**

We present an analysis that compares aortic morphological and clinical outcomes of 36 patients, all treated with endovascular uncovered stents implantation preceded or not by stent-graft implantation, or surgical treatment in the context of complex treatment of type A or type B aortic dissection.

**Methods:**

Between 2014 and 05/2018 our team treated 36 patients with acute aortic dissection and end-organ ischemia due to true lumen compression. All clinical and periprocedural data were obtained prospectively, followed by a retrospective analysis. The case series aim is to show induction of aortic remodeling by depressurization of the false lumen and increasing the size of the true lumen by non-covered stents implantation in the aorta and its affected side branches. Secondary endpoints were survival, branch patency, true lumen and false lumen size evolution.

**Results:**

Results from the diameter of both lumens measured by computed tomography angiography (CTA) before and at least 1 year after the treatment showed statistically significant differences, patent stents, as well as symptomatic improvement in all patients. Both aorta-related and general mortality in this complex group of patients was 0%.

**Conclusions:**

The concept of redirection of flow in aortic dissection with non-covered stents was safe, led to positive aorta remodeling and resulted in excellent survival rate.

## 1. Introduction

Acute aortic dissection is the most catastrophic and life-threatening disorder affecting the aorta [1]. The incidences of death in patients having type A or type B dissection repair remains high, despite the continuous improvements in the endovascular/surgical, medical management and imaging techniques [1, 2]. End-organ malperfusion presents as one of the most dismal complications of acute aortic dissection [3]. Malperfusion is defined as reduced blood flow to the arterial bed of a vital organ. A malperfusion syndrome occurs when the diminished blood flow results in ischemia and impaired function of the affected organ [3].

The incidences of end-organ malperfusion range from 16–34% and may involve any of the main arterial side branches [4]. The major causes of these unfavorable postsurgical results are malperfusion of the vital organs and persistent perfusion of the false lumen after open surgical or endovascular repair. According to published studies, an effective method to cope with this complex pathology is the hybrid procedure, which combines ascending aorta/aortic arch resection followed by distal endovascular stabilization of the descending thoracic aorta [1, 2, 3, 4].

We have observed a transformation in the management of complicated type B aortic dissection with the launching of new devices designed for thoracic endovascular aneurysm repair (TEVAR) [1]. Entry tear coverage with TEVAR and redirection of blood flow entirely through the true lumen proved to be the key features of the endovascular repair [5]. The 5-year survival rate for chronic type B aortic dissection, treated with medical therapy alone is as high as 60% to 80%, because it is observed that the disease progresses, despite the treatment and many patients develop serious complications. In this group of patients, around 59% of them have had a progressive aortic dilatation with a mean expansion rate of 1.7 ± 7 mm/year [6, 7]. Medical therapy should always be recommended for patients with uncomplicated type B aortic dissections, and TEVAR should be considered. On the other hand, for complicated type B aortic dissections, TEVAR is the gold standard for treatment [2]. Long-term data from the INSTEAD XL trial, a randomized trial of best medical management with or without endovascular stent grafting, show that TEVAR has a significant benefit between 2 and 5 years after the intervention in terms of all-cause mortality (0% *vs.* 16.9%), aorta-specific mortality (0% *vs.* 16.9%), and disease progression (4.1% *vs.* 28.1%) [7]. Even though TEVAR is currently the accepted method of treatment, it often does not provide good remodeling, which leads to aneurysm degeneration. To prevent possible late complications and to improve the remodeling rate, we adopted the “provisional extension to induce a complete attachment” (PETTICOAT) technique as routine procedural practice. This technique uses an additional bare metal stent, which is deployed distally to the TEVAR device during the primary procedure [8]. Nixon and Mossop [9], were the first ones to describe the usage of bare-metal stents in order to promote true lumen expansion after entry tear coverage with TEVAR. Nienaber *et al*. [9], described 12 cases (from a series of 100 patients), where persistent true lumen collapse was present after endograft coverage of the proximal entry tear only and were treated with additional bare-metal scaffolding stents [10]. This strategy proved to restore flow to malperfused branch vessels and induce positive aortic remodeling. The concept is termed Staged Total Aortic and Branch vessel Endovascular (STABLE) reconstruction and potentially avoids late complications of aneurysm change, repeat dissections, and rupture [11]. The results from the ongoing STABLE Trial, which evaluates the safety and efficacy of a composite endovascular system consisting of TX2 thoracic stent-grafts and distal bare-metal stents for the treatment of complicated type B aortic dissection in 40 patients, demonstrated favorable clinical and anatomical results [12].

We represent a single-center analysis that compares aortic morphological and clinical outcomes for 36 patients, all treated with endovascular uncovered stent implantation combined or not with stent-graft implantation or surgical treatment in the context of complex management of complicated by malperfusion syndrome type A or type B aortic dissection.

## 2. Methods and Results

Our center annually performs significant number of both surgically and endovascular intervention in patients with acute aortic dissections. We performed both retrospective analyses of prospectively collected data of our group to create this overview. The aim of the overview is to prove the safety and efficacy of this treatment strategy and to follow up the positive aortic remodeling, achieved by depressurization of the false lumen and resolution of the true lumen compression.

Between 2014 and 05/2018 our team treated 36 patients with acute aortic dissection and end-organ ischemia due to true lumen compression. The majority of patients (32) were men 88.8 %, 11.1% female. The mean age in the group was 53.39 ± 13.13 years. Most of the patients had multiple risk factors listed in Table 1.

**Table 1.** **Risk factors**.

Risk factors	% (N)
Arterial hypertension	94.6%
Dyslipidemia	75.1%
Smoking abuse	45.9%
Family history	21.6%
Diabetes	43.1%
Chronic kidney disease	75.5%

Our cohort consists of both Stanford type A and type B aortic dissection. Type A dissection had 12 patients (33.3 %) of the studied group while 24 (66.6%) presented with type B dissection. Three of the 12 (25%) patients having type A dissection had previous aortic surgery, but significant end-organ damage was present due to compression of the true lumen from the false. They have received a simple proximal treatment (implantation of short surgical graft with securitization of the most proximal tear) without partial or total arch replacement. The majority of these cases were done in other hospitals and came in the acute/subacute phase after the primary surgical intervention. Despite the previous ‘proximal’ surgical correction of the disease, most of the patients in our group, presented with acute malperfusion syndrome in different vascular territories. We have evaluated the organ hypoperfusion clinically and with the help of imaging modalities and laboratory findings. The lactate levels were measured and they were severely elevated in all of the patients (≥6 mmol/L), more so the patients in these group were in the acute phase with symptoms onset <2 days prior to the presentation in our hospital. Since, the clinical findings and the acute onset, our main strategy was to centralize the blood flow in the aorta with the use of non-covered stents, which we have telescoped into the surgical/endovascular prosthesis or/and stenting of the main side branches responsible for the end-organ hypoperfusion, again with the help of non-covered stents. The time from the onset of the malperfusion syndrome evidences to treatment was 2–14 days. The main goal is to achieve malperfsion resolution as soon as possible, in order to avoid the static true lumen compression, which is typical for these chronic and complicated cases. Two of the patients, received elective additional open stents in the arch as prolongation of the surgical intervention (a version of the petticoat strategy used routinely in the pure endovascular group). Surgical debranching techniques were used in 4 patients. Totally endovascular repair was performed in 1 patient [13]. Type B aortic dissection with malperfusion was successfully treated in 24 patients. Our positive results could be explained with the fact, that in most of the cases the malperfusion was corrected with aortic stents implantation targeting not only to centralize the flow in the true lumen, but also to achieve restoration of the flow into the true lumen of the side branches, involved in the malperfusion phenomenon and rarely requiring recanalization and stent implantation in these affected branches. The extension of the dissection in the aortic branches is shown in the table (Table 2).

**Table 2.** **Patients characteristics**.

	% (N)	
Type A	33.3% (12)	
Type B	66.6% (24)	
Debranching prior endovascular procedure	10.8% (4)	
Prior surgical repair in type A	8.3% (3)	
Affected aortic branches		
	Affected side branches	Additional side branch stenting
Brachiocephalic trunk	22.2 % (8)	8.33 % (3)
Carotid artery	13.8% (5)	8.33 % (3)
Subclavian artery	44.4% (16)	13.89 % (5)
Coeliac trunk	22.2% (8)	0% (0)
Mesenteric artery	19.4% (7)	5.55% (2)
Renal artery	Right renal artery-27.8% (10)	5.55% (2)
	Left renal artery-36.1% (14)	
Iliac artery	Right iliac artery-30.56% (11)	8.33% (3)
	Left iliac artery-41.67% (15)	

### 2.1 Procedure

**Fig. 1.**
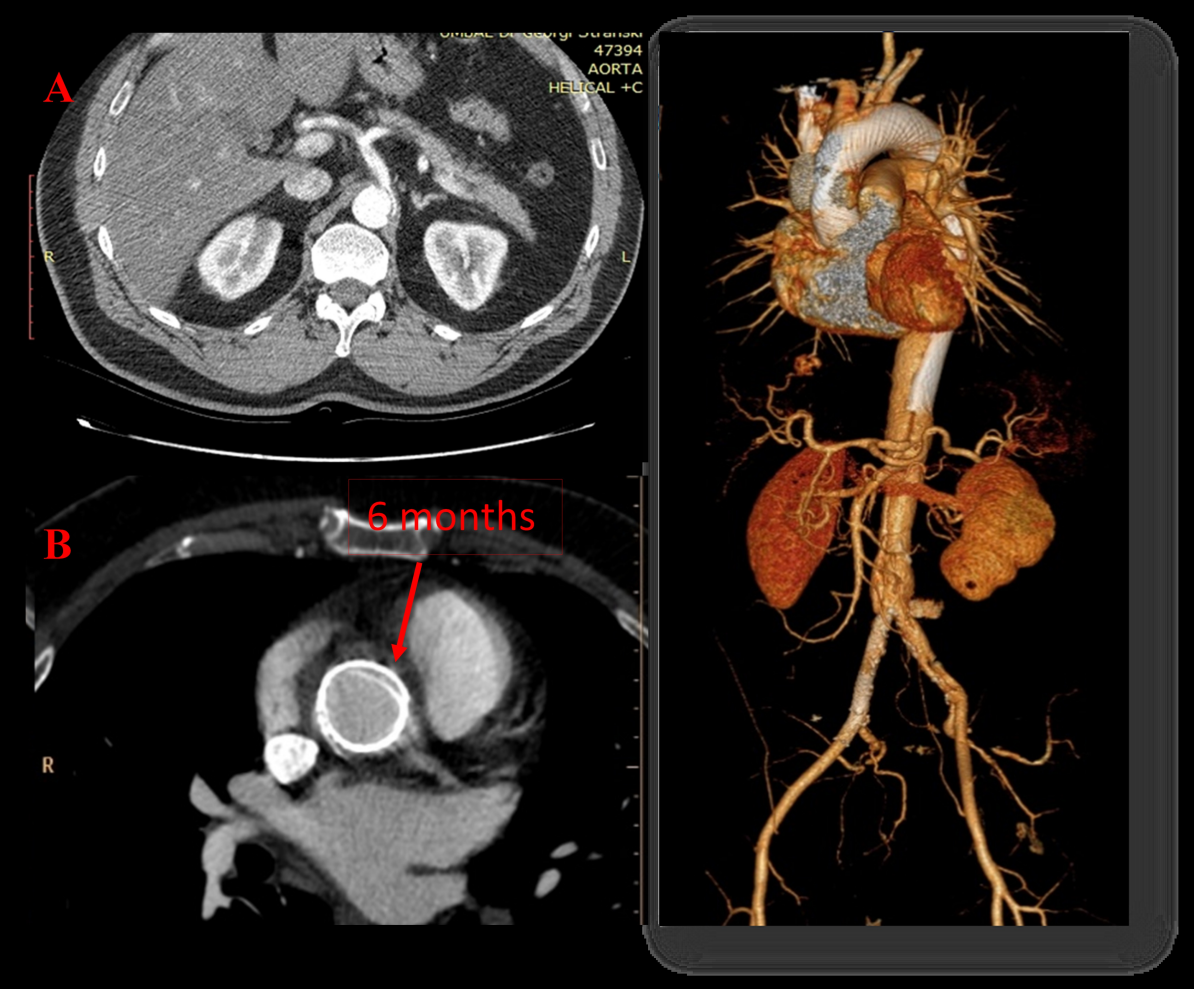
**Implantation of a non-covered multilayer Cardiatis MFM stents (Cardiatis, Isnes, Belgium)**. A 48-year-old male with aortic dissection Stanford type A extending from the aortic arch after the ostium of the brachiocephalic trunk to both iliac arteries (A). We implanted two overlapping MFM (Cardiatis) 35/200 mm into the aorta from the coronary arteries to renal arteries + two nitinol Protégé stents in the compressed right iliac true lumen. Computer tomography (CT) at 6 month showing excellent ascending aorta remodeling with progressive passivation and healing of the false lumen and complete centralization of flow into the true lumen (B).

**Fig. 2.**
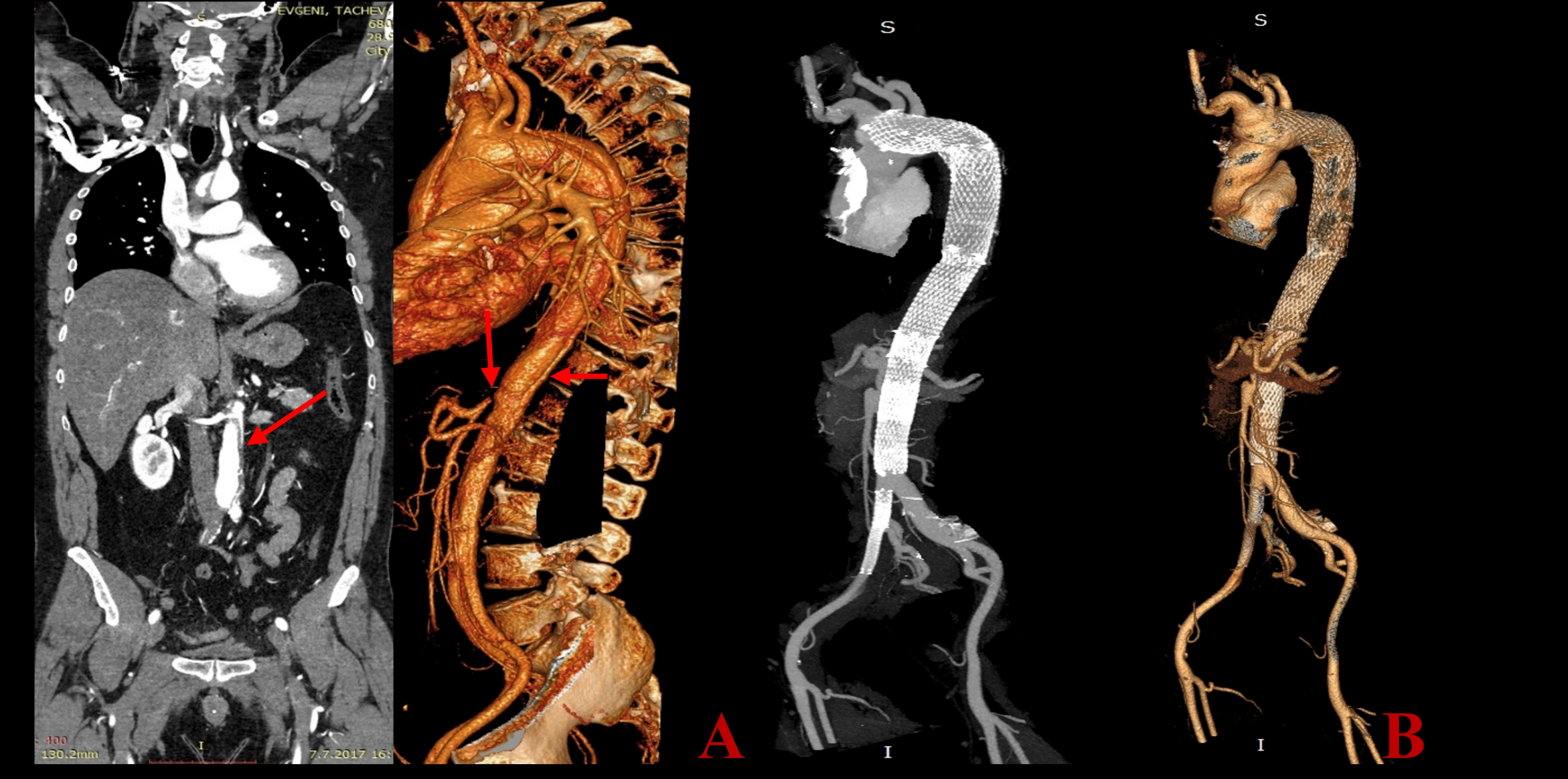
**A 55 years old male, presented with type A aortic dissection (AD), treated with an emergent surgery-resection of the ascending aorta, partial arch repair and implantation of a graft**. Postoperative critical medical condition (ileus, acute renal failure, right leg hemiparesis) due to persisting significant compression of the true lumen in the thoracic and abdominal aorta with critical visceral and peripheral ischemia. We did an emergent endovascular procedure with implantation of five overlapping Sinus XL stents from the aortic arch to the abdominal aorta and two Everflex stents in the iliac arteries. A–preoperative CT construction. Arrows showing the site of the malperfuusion. B–1-year CT scan follow-up results - Centralization of the blood flow in the true lumen with a patent blood flow in the supraaortic and visceral arteries.

We obtained informed consent for the endovascular procedure from all of the patients. Vascular access of choice was femoral for the aortic stent implantation and radial/brachial for pigtail catheter placement and visceral and renal stents implantation. In all type B dissections left radial approach was used for pigtail catheter insertion and positioning in the ostium of the left subclavian artery as an anatomical landmark during the implantation of the proximal stent-graft. General anesthesia was chosen in 5 patients due to clinical conditions, local anesthesia plus sedation was the method of choice in the other 31. The average X-ray time was 14.3 min. The average amount of contrast used was 151 mL. Different stents were chosen for the patients with sizes following the proximal and distal reference diameter of the healthy aorta in a 1:1 ratio. The Valiant Thoracic Stent Graft System (Medtronic Vascular, Santa Rosa, California, USA) was implanted in 15 patients in order to close the primary aortic intimal tear. In the total group of 36 patients 38 bare (non-covered stents) were implanted (20 aortic and 18 branch stents). In 13 of the patients non-covered Sinus-XL stent (Optimed, Ettlingen, Germany) was used and in the other 7 patients the stent of choice was non-covered multilayer Cardiatis MFM stents (Cardiatis, Isnes, Belgium) (Figs. 1,2).

**Fig. 3.**
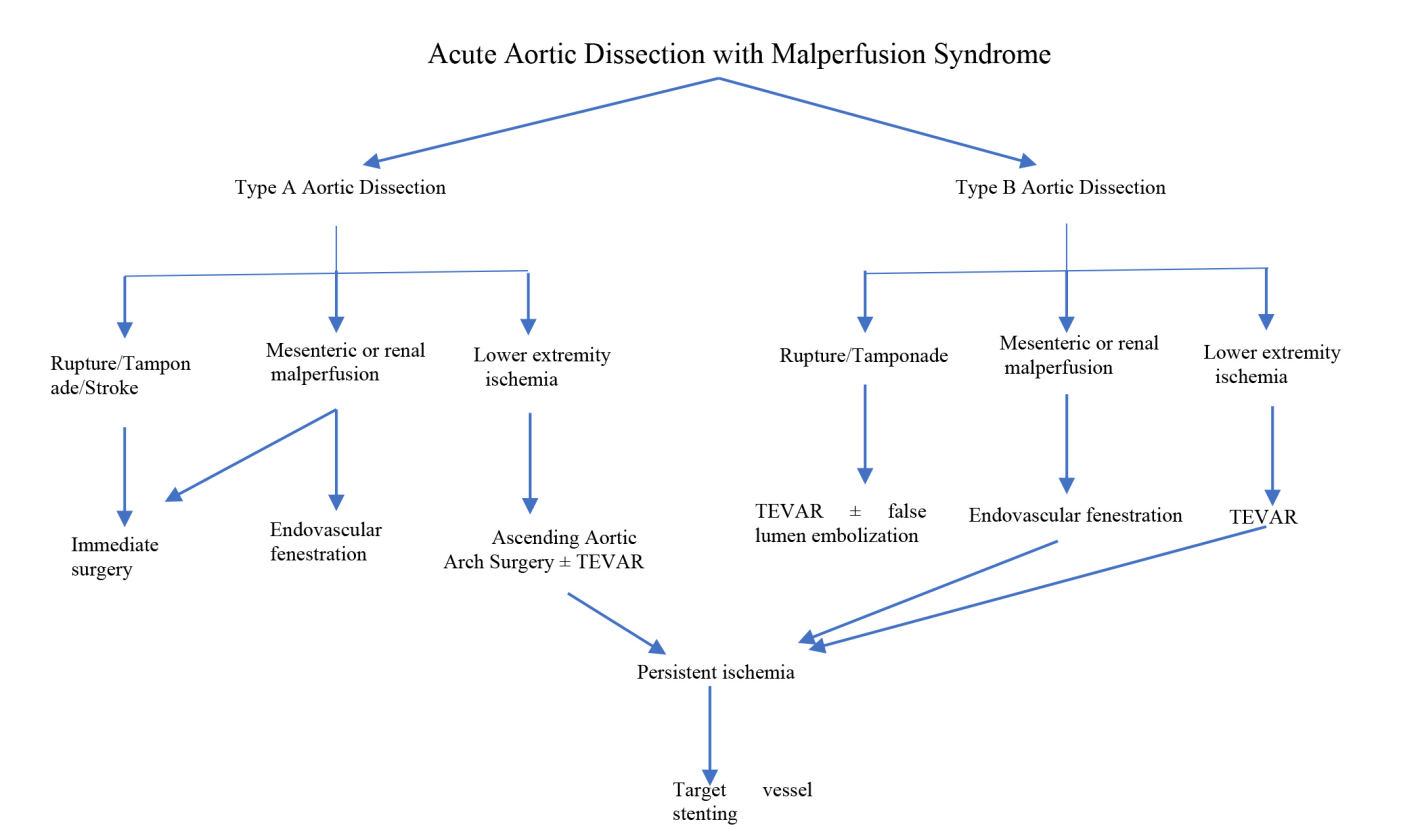
**The algorithm of our cardiovascular center for the treatment of AD and/with malperfusion syndrome**. TEVAR, Thoracic Endovascular Aortic Repair.

In 15 of the patients we managed to close the vascular access with Perclose ProGlide (Abbott Vascular 3200 Lakeside Drive Santa Clara, CA 95054, USA) closure device (in 12 patients two devices per patient were used and in 3 only one, according to the size of the vascular sheath). In one of the patients three ProGlide closure devices were needed in order to properly close the vascular access. In 3 patients the closure device of choice was Angio-Seal 8 Fr (St. Jude Medical, St. Paul, MN, USA). In the other 18 patients a primary surgical closure of the main vascular access site was adopted. Patients were followed up for major complications (access vascular site, renal impairment, aorta related, neurological). None in-hospital death or other major complication was observed in this very complex group of patients. Minor complications were vascular access site hematoma in 3 patients, one of which was successfully resolved surgically. The average ICU stay was 4,6 days (range 1 to 11 days), considering the fact that most of the patients were in critical condition, requiring prolonged monitoring and intensive care. Based on the pattern of anatomic obstruction, the aortic branches obstruction can be classified as static, dynamic or both. It can also, lead to persistent (static obstruction) or intermittent (dynamic obstruction) malperfusion of the affected organs [11]. In 14 patients, endovascular treatment was extended to stent implantation in the supra-aortic vessels or descending aorta branches with the purpose of resolving the underlying end-organ ischemia and improving run-off of the true lumen (Table 2). Also, we have used the molding balloon for 3 of the aortic stents. This is the algorithm we have used in the last 8 years in our cardiovascular center (Fig. 3).

### 2.2 Follow-Up

The renal function was estimated at admission and during the follow-up. At baseline, 28 of the treated patients were with signs of chronic kidney failure. We closely followed the creatinine values in 24 of the patients, who had involvement of the renal artery in the compressed true lumen zone. Significant improvement in the renal function was observed in all during the postprocedural period. The initial mean values of 177.43 mmol/L were decreased to 127.22 mmol/L at 6-month follow-up (*p* = 0.185) (Table 3). A small part of the group-4 patients were initially in anuria due to severe kidney malperfusion with restored diuresis post-procedurally in all of them. Moreover, the lactate levels were within normal limits prior to discharge.

**Table 3.** **Creatinine values**.

Creatinine values (mmol/L)	N	Mean	Median	SD	Min	Max	Percentiles	
Before procedure	36	177.43	93.00	133.24	61.00	750.00	79.00	111.50
During follow up	36	127.22	89.00	41.20	66.00	313.00	78.00	104.00

During the follow up, our team reevaluated and compared the antihypertensive medical therapy of the patients. At discharge, the mean number of antihypertensive medications were 3–5 (>55% of patients). We established a tendency of reducing the number of antihypertensive medications during the follow-up with 2–4 (>60% of patients). The probable reason is the decrease in vasoactive substance release due to restoration of the true lumen flow in the renal segment.

### 2.3 CT Follow-Up

**Fig. 4.**
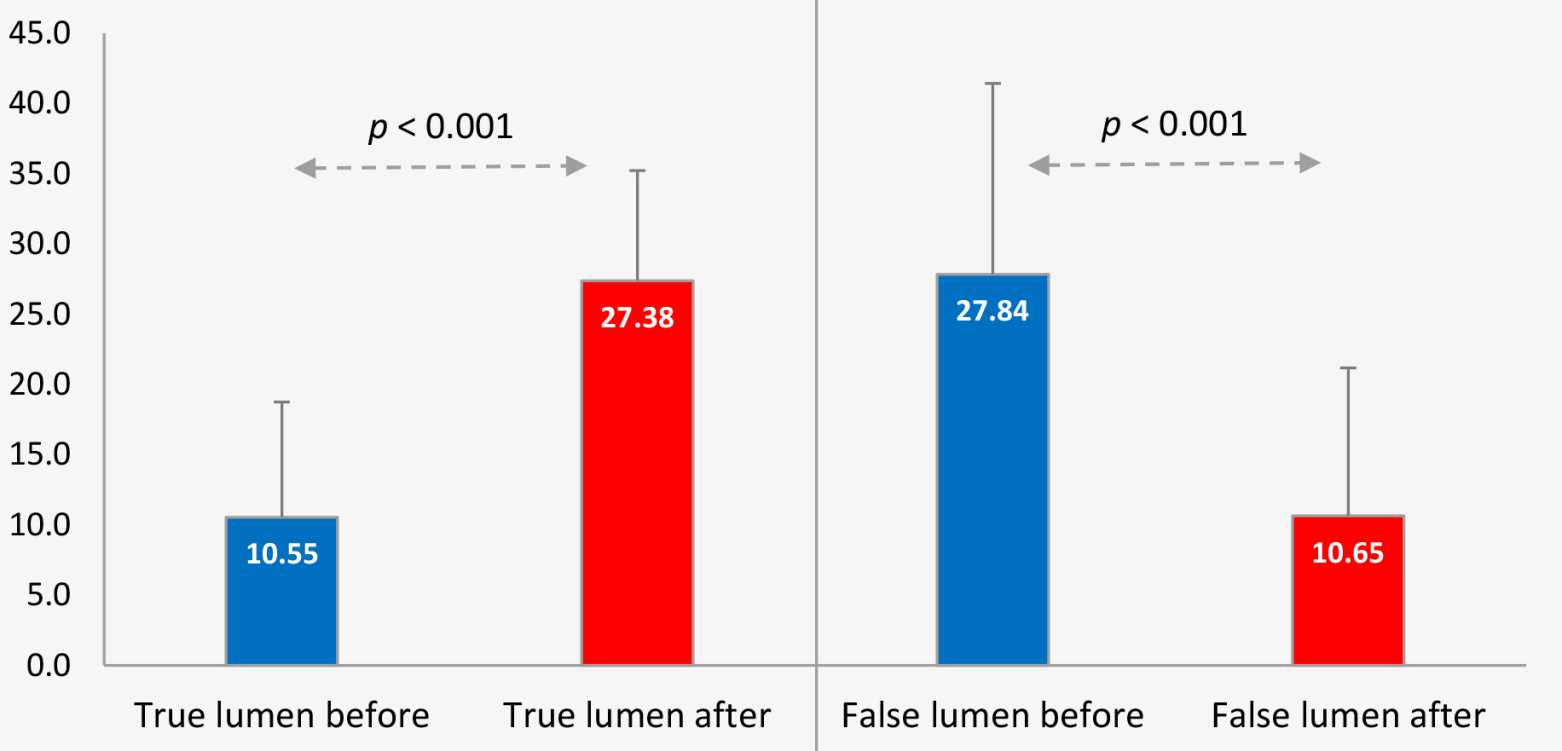
**True and false lumen evolution in the post-procedural period**.

The morphological analysis of the aorta includes localization of the intimal tears, compression zone, minimal diameter of the true lumen and maximal diameter of the false lumen before and after endovascular treatment. All patients underwent the 6 months follow-up. We present the results measured at the site of the most significant compression before intervention and at the follow-up (Table 4). From baseline to follow-up, the mean minimal true lumen along the aorta expanded significantly from 10.5 mm to 27.38 mm (*p *< 0.001) and mean maximal false lumen diameter decreased from 27.84 mm to 10.65 mm, corresponding to a significant decrease in the false lumen size (Fig. 4).

**Table 4.** **Diameter values of the true and false lumen before and 6 months after the procedure**.

Diameter of	Mean	SD
Minimal true lumen diameter before	10.55	8.2018
Minimal true lumen diameter after	27.38	7.854
Maximal False lumen diameter before	27.84	13.600
Maximal False lumen after	10.65	10.523

Measurements based on control computed tomography angiography (CTA) scan showed that the true lumen is decompressed in all stented zones with a statistically significant decreased size of the false lumen, as well as induced thrombosis (Table 4.1). Based on our previous experience, we used the elevated levels of D-dimer as a laboratory marker for thrombosis induction. The objective relief of the visceral ischemia was ascertained by laboratory tests in all patients–the marker was normalization of the lactic acidosis. Bowel ischemia was defined, according to the triad of abdominal pain, diarrhea and elevated serum lactate.

**Table 4.1.** **True and false lumen differences peri procedurally**.

	Differences				t	df	*p*
	Mean	SD	95% CI				
True lumen before and after implantation	16.83	8.22	–9.57	14.08	12.45	36	<0.001
False lumen before and after implantation	–17.19	–12.45	–13.04	–21.34	–8.40	36	<0.001

The follow-up CTA control showed that implanted aortic and side branch stents contributed to flow restoration in the true lumen, side branches and end-organ perfusion, thus preventing aortic rupture and end-organ ischemia. The side branches (stented or not) arising from the aortic stented area were all patent during the follow-up period. The restenosis rate (more than 50% stenosis) and target vessel revascularization, target lesion revascularization (TLR/TVR) rate for the stented side branches was 0%. We observed transient oozing in 9 patients with Proglide closure device. In one patient we detected a type III endoleak upon control examination, one-month post intervention. Other major complications were one pseudo aneurysm formation and one access site arterial stenosis. We had two cases of femoral artery thrombosis, which were treated endovascularly without any further complications. There was no death and no complications requiring limb amputation. Primary Proglide device failure occurred in 4 cases which were all tackled successfully with an implantation of additional Angioseal 8Fr closure device.

During the follow-up period all patients showed improvement of the symptoms and clinical status and we did not observe any midterm or late complications, no major adverse cardiovascular events (MACE) and no aorta related mortality.

## 3. Discussion

We present a group of patients all treated with endovascular uncovered stent implantation combined/or not with stent-graft implantation or surgical treatment in the context of complex treatment of type A or type B aortic dissection complicated by end-organ ischemia. Our case series shows that induction of aortic remodeling by depressurization of the false lumen and increasing the size of the true lumen by non-covered stents implantation in the aorta and its affected side branches is a safe method with excellent survival rate with both aorta-related and general mortality in this complex group of patients was 0%. The limitations of the study are that even though the complex nature of the disease our sample size is relatively small and there is a possibility of potential survival bias, due to the fact that some of the patients were transferred from other facilities.

Additional tears, critical true lumen compression, and true lumen obliteration with end-organ ischemia can be either life-threatening in the acute phase or compromise acute and chronic clinical outcomes after surgical/endovascular intervention of aortic dissection (AD). Since, malperfusion is one of the most problematic complications of AD (second leading cause of death in aortic dissection after rupture). Therefore, treatment directed to correct the malperfusion is crucial for the patient’s prognosis. Residual patent false lumen is an independent predictor of long-term mortality and aortic events in both type A and type B aortic dissections. The incidence of malperfusion varies in the literature from 10–33% and can occur with both acute type A and type B dissection [13, 14, 15, 16]. Malperfusion syndrome gives a rise to an inflammatory cascade, resulting from end-organ ischemia. Endothelial injury and impaired membrane integrity in the ischemic tissue result in neutrophil activation and subsequent generation of free radicals. In ischemic settings, there is higher production of myeloperoxidase and also increased complement consumption in the affected organs. Studies have demonstrated upregulation of the tumor necrosis factor-alpha (TNF-α) and interleukin-1 (IL-1), resulting in higher adhesion of the cells and increased production of molecules that leads to extravasation of leucocytes into the malperfused tissue and there by leading to the release of even more cytokine. There are two mechanisms responsible for malperfusion to occur–from static and dynamic obstruction. When the ventricle contracts, it produces a fluid column that travels down both the true and the false lumen. According to the Laplace law, there is ectasia of the false lumen because it lacks elastin and cannot take in the generated wall tension. The pressure difference between the false and the true lumens could lead to movement of the mobile intimo-medial septum - bulging into the ostia of the aortic branches and to obliterate them. This is the main mechanism behind the transient or persistent static obstruction. Static obstruction cannot be managed with the help of medications, it will always require interventional correction. On the other hand, for the dynamic obstruction to occur there are two distinct etiologies. If branch vessel perfusion is maintained by the true lumen, insufficient flow through it may lead to hypoperfusion. The other mechanism of dynamic obstruction reflects the mobility of the intimal flap. It happens when the false lumen prolapses into a branch vessel ostium and the flow is dynamically compromised. In the acute and subacute fazes, dynamic obstruction is responsible for approximately 80% of malperfusion syndrome cases. This type of obstruction, is intermittent and the number of events of dynamic obstruction can be reduced by centralization of true lumen flow by endovascular aortic stent implantation, because it depends on changes in the blood pressure and hemodynamic forces. In some cases, this pathologic mechanism can be controlled with medications [17, 18]. As we can see, the proper distinction between the two mechanisms of branch-vessel compromise is important because they necessitate different modes of treatment. The static narrowing can be treated locally with an intravascular stent, as in any other elastic stenosis, while the dynamic obstruction will not respond to treatment with an endoluminal stent. Treatment in these cases should be directed at restoration of the flow in the aortic true lumen [18, 19]. The dynamic obstruction can be managed by two distinct endovascular solutions–coverage of the entry tear by TEVAR and/or fenestrating the intimal flap [20, 21]. The major factor contributing to the successful outcome after acute malperfusion in aortic dissection is the rapidity with which blood flow is restored to the ischemic organs or limbs. The treatment should be done as fast as possible, prior to the irreversible static obstruction and ischemic damage. In the study reported by Beregi *et al*. [22], they demonstrated a significantly lower mortality rates compared to the usually reported, because they performed the endovascular procedure for correction of the malperfusion more rapidly and with priority to other interventions.

According to the latest guidelines, for patients with type A AD, urgent surgery is recommended, but if the dissection is complicated by malperfusion syndrome, a hybrid approach is preferred [2]. Results from a study by Kamman* et al*. [23], showed that surgical delay is associated with lower mortality rates. They recommended an endovascular strategy as a first step for the relief of branch vessel obstruction and then urgent aortic repair. This approach avoids the inflammatory response associated with prolonged cardiopulmonary bypass in the settings of end-organ ischemia [23]. An interesting alternative option for patients with type A AD is proposed by Rupprecht *et al*. [24]. They inserted an uncovered stent into the aortic arch in order to cope with the possible risk for later complications, because in these cases the aortic arch remains untreated and free for future formation of aneurysm or retrograde aortic dissection [24]. Aortic arch complications and persistent patent distal false lumen are problematic late complications after surgery for type A AD, which could require future endovascular repair with intimal fenestration and stent-graft implantation [21]. As we can see, there are two different strategies for the management of patients presenting with type A AD. The proximal strategy adopts the method of immediate surgery in order to close the more proximal entry tear. On the other hand, we can delay the surgery in favor of early endovascular treatment of malperfusion as a first step toward clinical stabilization. We believe that, in the absence of serious cardiac complications, endovascular correction of malperfusion prior to the surgery is the better approach. The proximal strategy carries a high periprocedural risk, leads to future reinterventions in more than 50% of the patients, because of persistent ischemia, making it more costly to the healthcare system. Treating the malperfusion syndrome, prior to the surgery has been proven to be more successful and to give a better prognosis for the patient with fewer future reinterventions and lower mortality rates [20].

According to some published analyses, an endovascular individualized approach for treating acute complicated type B aortic dissection, seems to have a beneficial effect compared with an open surgery [25, 26, 27]. The goal of TEVAR is to achieve the complete elimination of antegrade flow into the false lumen by closing the primary tear with a covered stent, placed into the true lumen. This leads to true lumen expansion and reduces false lumen blood flow with subsequent thrombosis and shrinkage of the false lumen, which has been termed aortic remodeling [28]. TEVAR usually leads to positive aortic remodeling, but this is mostly limited to the aortic segment covered by the stent graft itself. Frequently, maneuvers and interventions for additional stabilization and expansion of the proximal and distal true lumen are needed to provide better organ perfusion and to induce favorable aortic remodeling. Complicated AD after surgical or endovascular treatment of both type A and type B AD, can require further intervention to decompress critical compression of the true lumen, restore blood flow in side branches or in cases of life-threatening organ ischemia. The go to treatment of choice for these cases can be the endovascular treatment with non-covered stent implantation because debranching providing sufficient landing zone for covered stents implantation is not always possible or is too risky [29, 30]. During the last years, several new stent technologies with dense mesh manage to achieve the idea of flow centralization (flow diversion, flow modulation) [31]. The aims are***—***covering of the proximal entry tear, depressurization of the false lumen, leading to its reduction in size and subsequent thrombosis, redirection of the blood flow towards the true lumen both in the aorta and its branches***—***all of these lead to a favorable “aortic remodeling” process in the follow-up.

Our team managed to achieve a statistically significant reduction of the diameter of the false lumen in this cohort of patients with complicated type A and B aortic dissections with a mean maximal false lumen size reduced from 27.84 mm to 10.65 mm. This is probably the cornerstone of the high survival rates that we report. As reported by Song *et al*. [32], patients with false lumen diameter > or = 22 mm show higher event rate (aneurysm or death) than others (*p *< 0.001). In our case series, all patients had 100% survival and symptom improvement. We did not observed any aortic rupture or descending aorta replacement. There were no neurologic complications (e.g., paraplegia, transient ischemic attack, or stroke). All patients are alive and well at the time of this report.

## 4. Conclusions

The concept of redirection of flow in complex cases of aortic dissection complicated by malperfusion syndrome with non-covered stents implantation in the aorta and side branches was safe, led to positive aorta remodeling and resulted in an excellent survival rate. The goal to achieve centralization and restoration of the blood flow in the true lumen of both the aorta and the affected side branches is the most probable explanation of our highly positive results.

## Data Availability

All data generated or analyzed during this study are included in this published article.

## Abbreviations

AD, aortic dissection; TEVAR, thoracic endovascular aneurysm repair; CTA, computed tomography angiography; MACE, Major adverse cardiovascular events.
